# Supplementary figures and images for: ProAKAP4 Semen Concentrations as a Valuable Marker Protein of Post-Thawed Semen Quality and Bull Fertility: A Retrospective Study
Source: Vet Sci. 2022 May 6;9(5):224. doi: 10.3390/vetsci9050224 (PMC9144616; doi:10.3390/vetsci9050224)

Figure S1: The whole Western blot figure

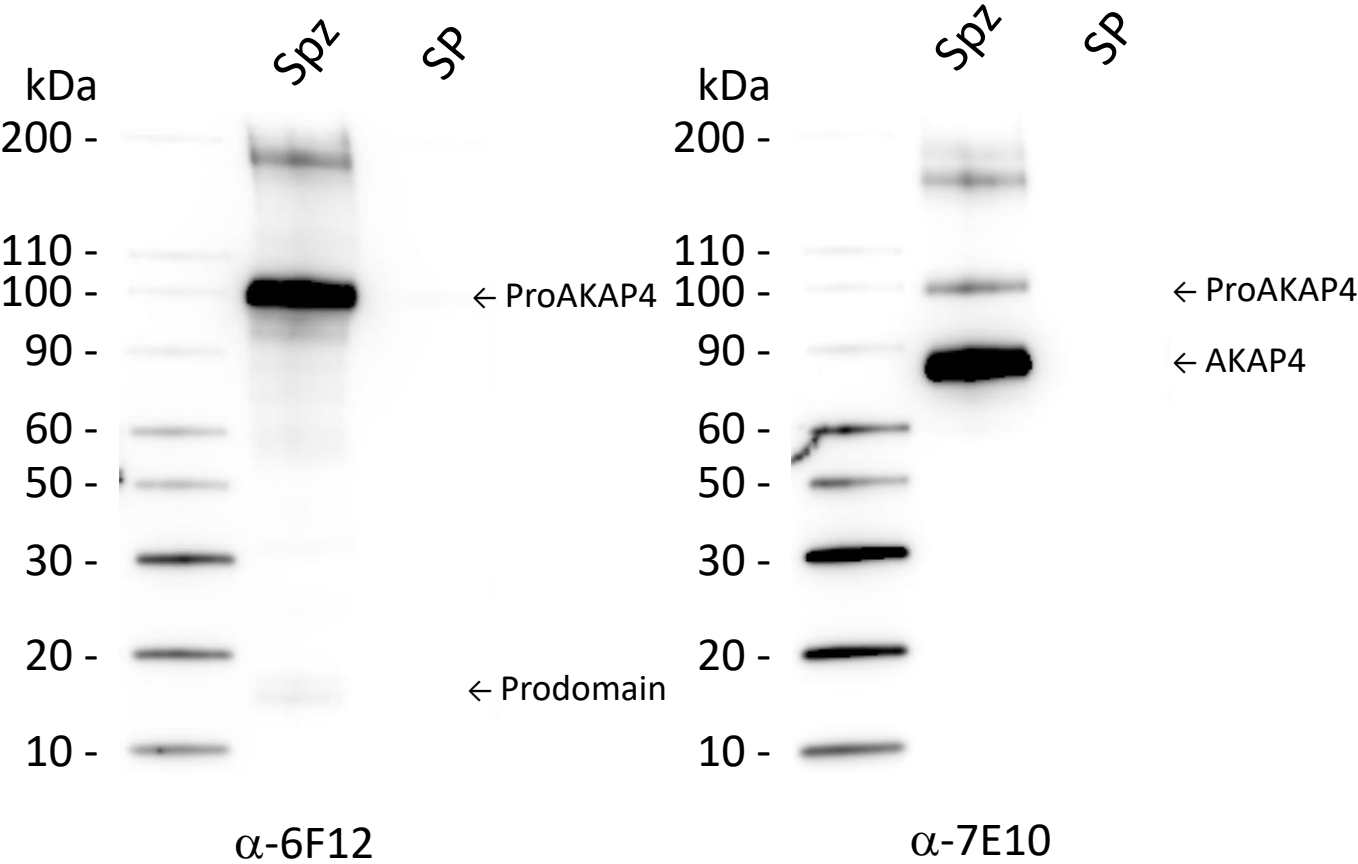

Uncropped Western blots

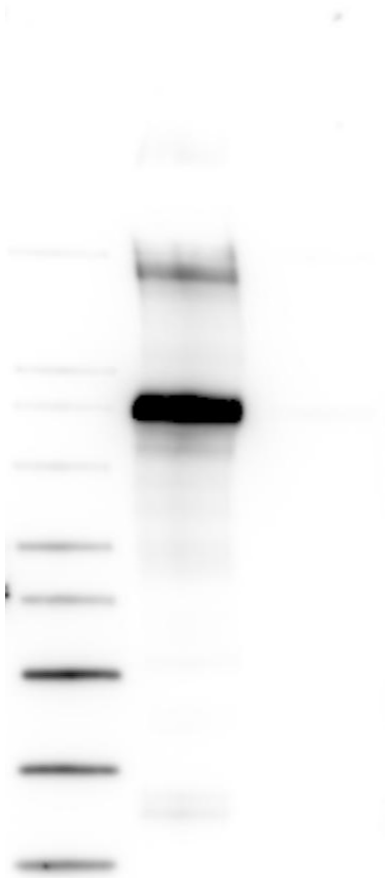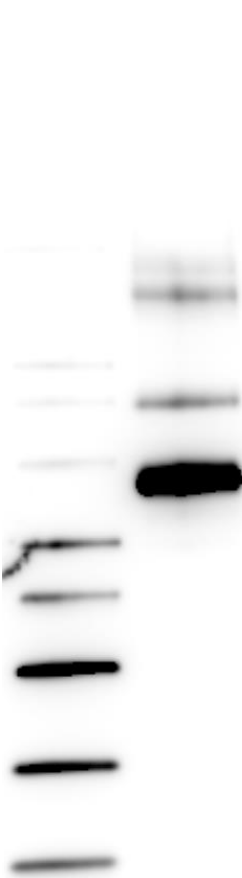

Supplement: Supplementary file 1 [file vetsci-09-00224-s001.zip › vetsci-1663107-supplementary.pdf]
